# Fabian Deliverances on Sanatoria

**Published:** 1913-05-24

**Authors:** 


### The Hospital

The Workers' Newspaper of Administrative Medicine and Institutional Life, Administration, National Insurance and Health.

No. 1403, Vol. LIV | SATURDAY, MAY 24, 1913. | PRICE ONE PENNY.

### FABIAN DELIVERANCES ON SANATORIA.

No social reformer nowadays can leave medicine out of his purview, and certainly none does. Accordingly, when, last month, Mr. Bernard Shaw and Mr. Sidney Webb began to publish a magazine called *The New Statesman*, we looked out for signs of the continuance therein of that criticism of medical affairs of which both have been liberal in the past. In the fourth number there duly appeared an article on the decline of tuberculosis, marked by the usual leading feature of the Fabian case against present-day medicine—namely, top-heaviness from overstating. What the writer, who signs himself "Lens," runs a tilt at is Dr. Archdall Reid's teaching with regard to protective racial evolution against disease—that "every race is subject to a disease in inverse proportion to its former sufferings, because the disease weeds out the susceptible from generation to generation until at length there is produced a race the members of which are all practically immune." Now this impressive theory, which everyone allows was well argued by its author, is disposed of in three or four sentences. The real facts of the epidemics among unaccustomed South Sea Islanders will probably never be known; so much for protective evolution against the exanthemata. Alcoholism makes great ravages in modern industrial Italy: so much for protective evolution against drunkenness. As regards tuberculosis, there is simply no evidence at all for protective evolution; and no first-hand student of tuberculosis believes in it.

Well, it must just be pointed out that a good number of medical men in South Africa believe that if you have a consumptive patient of mixed black and white blood, the favourability of the prognosis, other things being equal, varies directly as the amount of the latter. And in one of the articles in a recent number of the *Journal of the American Medical Association*, we noticed a statement by a medical man that he had never seen a negro recover from even incipient tuberculosis. These are instances of first-hand study by observers who, being in countries populated by races of African and races of European extraction, are very well placed to judge of the comparative racial incidence of tuberculosis. Let us not, however, like the gentleman we are noticing, take never a look at the other side of the shield. Many—among them Sir William Osler—think that environment will wholly account for the large tuberculous mortality among, say, North American Indians and negroes, races whose period of close contact with the tubercle bacillus cannot be reckoned many generations. But there are others who have given good reasons against such a view; and, as regards the particular instances we have mentioned, we do not think any medical man could say of European slum dwellers what the American physician said of negroes—namely, that he had never seen even an incipient case get well. But surely the environment is no worse in the negroes' case.

The fact is, these not settled questions, with which medicine abounds, just because (our Fabian friends might have us on the hip here) it is pursued primarily as a therapeutic art, and not as a branch of knowledge—these questions are much too large to be pronounced on in a few lines. That surely is axiomatic. We hope it is not impolite to speculate on a possible motive for leading "Lens" to disregard the axiom. Now, the "dead hand of heredity" may be just as repugnant to the social reformer as it is to the theologian. We have all heard of the pother raised by the teetotallers when they became alive to the determinist interpretation that may be put upon Dr. Reid's teaching about alcoholism. If the race will automatically free itself of drunkards, the temperance advocate's occupation's gone. If it is also automatically freeing itself of consumptives, so is one of the Fabian's best arguments against a society which rears itself upon the plinth of the poor, crushing them into disease with its weight. . . . We hope it is not impolite to suspect a trifle of subconscious mental bias here. We are emboldened to say as much because his parting shot is—deliberately mixed metaphors are a literary device left timidly untried—of the same complexion. "The sanatorium does not alter the bio-chemical facts of its patients; and, in short, it only 'cures' those who are destined, in any case, to cure themselves." The first clause is none of the clearest; it sounds like the sort of thing that gets put down when the writer is near the end of his task and pressed for time. But the second surely means that sanatoriums have no therapeutic value at all. Well, many people, some of them medical men, have a habit of saying this. But the verdict of events is otherwise; and it is strange how very few who fall ill of tuberculosis refuse the hygienic-dietetic treatment. However, again, in proportion to the inefficiency of sanatoriums so much the greater is the case for prevention—for better housing, free-breakfast tables, and so forth. We are possibly wrong, and there may be no prejudice here at all. The regularity of Fabian sentiment and argument may not be that of antecedent and consequent. But it is there.

